# Contrast-enhanced mammography for surveillance in women with a personal history of breast cancer

**DOI:** 10.1007/s10549-024-07419-2

**Published:** 2024-07-04

**Authors:** Julia Matheson, Kenneth Elder, Carolyn Nickson, Allan Park, Gregory Bruce Mann, Allison Rose

**Affiliations:** 1 The Royal Melbourne Hospital, Grattan Street, Parkville, Australia; 2 Department of Surgery, The University of Melbourne, Parkville, Australia; 3 Daffodil Centre, The University of Sydney, a joint venture with Cancer Council New South Wales, Sydney, Australia; 4 Melbourne School of Population and Global Health, The University of Melbourne, Parkville, Australia; 5 The Royal Women’s Hospital, Flemington Road, Parkville, Australia; 6 Department of Radiology, The University of Melbourne, Parkville, Australia

**Keywords:** Breast cancer, Surveillance, Mammography, Contrast, CEM

## Abstract

**Purpose:**

Women with a personal history of breast cancer have an increased risk of subsequent breast malignancy and may benefit from more sensitive surveillance than conventional mammography (MG). We previously reported outcomes for first surveillance episode using contrast-enhanced mammography (CEM), demonstrating higher sensitivity and comparable specificity to MG. We now report CEM performance for subsequent surveillance.

**Methods:**

A retrospective study of 1,190 women in an Australian hospital setting undergoing annual surveillance following initial surveillance CEM between June 2016 and December 2022. Outcome measures were recall rate, cancer detection rate, contribution of contrast to recalls, false positive rate, interval cancer rate and characteristics of surveillance detected and interval cancers.

**Results:**

2,592 incident surveillance episodes were analysed, of which 93% involved contrast-based imaging. Of 116 (4.5%) recall episodes, 40/116 (34%) recalls were malignant (27 invasive; 13 ductal carcinoma in situ), totalling 15.4 cancers per 1000 surveillance episodes. 55/116 (47%) recalls were contrast-directed including 17/40 (43%) true positive recalls. Tumour features were similar for contrast-directed recalls and other diagnoses. 8/9 (89%) of contrast-directed invasive recalls were Grade 2–3, and 5/9 (56%) were triple negative breast cancers. There were two symptomatic interval cancers (0.8 per 1000 surveillance episodes, program sensitivity 96%).

**Conclusion:**

Routine use of CEM in surveillance of women with PHBC led to an increase in the detection of clinically significant malignant lesions, with a low interval cancer rate compared to previous published series. Compared to mammographic surveillance, contrast-enhanced mammography increases the sensitivity of surveillance programs for women with PHBC.

## Introduction

Surveillance breast imaging is important for women with a personal history of breast cancer (PHBC), as they have an increased risk of subsequent in-breast malignancy, and the cancers are on average higher stage than those detected in a general screening population [1, 2]. Early detection of subsequent cancers is associated with improved survival [1, 3, 4]. An interval cancer rate (cancers diagnosed between surveillance episodes) of 3.6 per 1000 mammographic screens has been reported in women with a PHBC compared to 1.4 per 1000 screens for women without PHBC [1].

Annual mammography (MG) is the standard surveillance imaging for women with PHBC [5, 6]. Supplemental ultrasound (US) may be used to increase surveillance sensitivity, however, it has low specificity [7–9]. Magnetic Resonance Imaging (MRI) yields increased cancer detection and lower interval cancer rates [10–12]. MRI is often considered in women with a cancer diagnosis before age 50, those with high mammographic density (MD) or with genetic predisposition, but is expensive with limited accessibility and not recommended for routine surveillance [13, 14].

Contrast-Enhanced Mammography (CEM) shows promise in screening and diagnostic settings with sensitivity approaching that of MRI and comparable specificity, without the resource constraints of MRI [15]. CEM combines digital mammography with intravenous injection of iodinated contrast to provide low energy images which are equivalent to two dimensional (2D) MG and recombined contrast images with information about lesion perfusion [18, 19].

CEM was introduced at the Royal Women’s Hospital in 2015 and the Royal Melbourne Hospital in 2018. From late 2018, CEM without supplementary US became the default surveillance modality for suitable, consenting patients with PHBC, with the expectation of improved outcomes compared to the previous standard of MG with or without US. We previously reported the first surveillance episode for 1,190 patients, demonstrating higher sensitivity and comparable specificity to MG, concluding CEM was an acceptable surveillance imaging modality [16].

We report subsequent outcomes for this cohort until the end of 2022, including cancer diagnoses, the contribution of contrast to cancer diagnoses, and interval cancer rates.

## Materials and methods

### Patient cohort

Hospital medical records of the previously identified cohort of women with PHBC who received their first CEM surveillance episode between June 2016 and October 2020 were reviewed [16]. All women having surveillance were re-offered CEM unless there was a contraindication such as renal impairment (eGFR < 30 ml/min) or contrast allergy.

### Surveillance imaging

Any surveillance imaging results following first surveillance CEM were recorded: this was CEM for the majority of women, but for some included MG with or without US, US alone, and MRI.

CEM was performed using a Hologic 3 Dimensions unit (Hologic, Danbury, Connecticut, USA). Patients were administered 100mls of Omnipaque™ 350 (Iohexol; GE Healthcare) intravenously, through a 20-guage cannula using a power injector, at a rate of 3 mL/sec. 2 min after the contrast injection was completed, the patient was positioned. Mammographic imaging was usually performed in “Combo Mode” with rapid low energy (26–30 kVp), high energy (45–49 kVp) and tomographic images interleaved. This provided 2D and 3D images and recombined contrast-enhanced images, with low energy images interpreted as the 2D MG component. Mediolateral oblique and craniocaudal views of each breast were obtained. The imaging window was from 2 to 8 min. Postprocessing with a recombination algorithm provided an iodine (C +) image that highlighted the areas of contrast enhancement. The low keV, tomographic, and the C + images were co-registerable and stored in PACS for reporting. Images were reported by at least one specialised breast radiologist. Adverse events, including contrast reactions with details on severity and outcomes, were captured in medical records.

### Recalls

Recall was defined as “any intervention instigated on clinical or radiological grounds arising from a surveillance episode”. Contrast enhancement above background was reported as requiring recall for further assessment. Each recalled case was reviewed to determine whether the recall was due to findings on 2D/3D, or only due to findings on Contrast (‘contrast-directed’). Interventions included targeted US, problem-solving MRI, early review CEM (usually at 6 months), percutaneous image-guided biopsy and excisional biopsy. As CEM-guided biopsy was not available in Australia at the time of this study, biopsy was directed by stereotactic MG or US if the lesion was identified with certainty, or MRI for contrast only lesions. A clip was deployed after all biopsies, then MG or CEM performed to confirm concordance. Recalls were classed as true positive (TP) where the final histopathology was invasive cancer or ductal carcinoma in situ (DCIS); all other recalls were classed as false positives (FP).

### Data collection

Data recorded included automated MD measurement (VOLPARA Health Technologies Limited, Wellington, New Zealand) and the degree of background parenchymal enhancement (BPE), initially graded adapting the BI-RADS classification of BPE for MRI and then according to the CEM BIRADS lexicon released in 2022 [17, 18]. Lesion classification was based on the radiologist report including the type of lesion, whether it was identified on 2D alone, C + images alone, or both 2D and C + . Cases reported as minimal signs’—where with the knowledge of enhancement a lesion could be identified on 2D images but was unlikely to have been identified without contrast-were grouped with cases identified on C + images alone for analysis as “contrast-directed recalls”. Further imaging, biopsy modality, histopathology results and treatment details were documented.

Reasons for patients ceasing surveillance imaging through the hospital service were recorded, including transfer to primary care (usually minimum 5 years post diagnosis), bilateral mastectomy, development of metastatic disease, and death.

Interval cancers were defined as invasive cancer or DCIS detected by physical examination and/or symptoms within 12 months of a normal surveillance episode. Cases of chest wall recurrence (on the side of previous mastectomy) or metastatic progression were not considered interval cancers.

Histopathological details of index cancers for the entire cohort were recorded. Triple negative breast cancer (TNBC, immunohistochemistry estrogen receptor (ER) and progesterone receptor (PR) expression of < 1% and no amplification of human epidermal growth factor receptor 2 (HER2)), and ‘ER-low positive’ cancers (ER 1–10%, PR negative, HER2 not amplified) were grouped for analysis [19–21].

### Data analysis

Data was tabulated in aggregate form with statistical tests applied using Stata 15.0 [22]. Analysis focused on surveillance rounds following first surveillance CEM (incident rounds). Two-sided Fisher’s Exact tests were applied to tabulated data, and t-tests were used to compare distributions between groups. The association of incident cancers with baseline factors was assessed using hazards models, applying log-rank tests of equality to compare hazards between groups.

For positive predictive value (PPV) calculations, PPV1 was defined as the positive predictive value of any recall and PPV3 was the positive predictive value of recalls resulting in biopsy. Cancer detection rate (CDR) refers to invasive cancer and DCIS, unless otherwise specified.

## Results

### Patient cohort

The cohort comprised 1,190 women with PHBC and at least one CEM for post-treatment surveillance (Table 1). Index cancers were invasive for 999 (84%) and DCIS for 191 (16%) patients and the most common invasive tumour subtype was ER/PR + HER2- (79%). Most patients had received breast conserving surgery (BCS, 81%). The median age at index cancer diagnosis was 55 years (range 23–89).

**Table 1:** Baseline characteristics of study cohort at first surveillance CEM [16]

Number (No.)	1190
INDEX CANCER	
Age (mean, median (IQR) (range))	55, 55 (49–62) (23–89)
Time since surgery (months) (mean, median (IQR) (range))	45.5, 36 (15–61) (2–235)
Index pathology (No. (%)) DCIS Invasive cancer	191 (16.1) 999 (83.9)
Index invasive tumour subtype (No. (% invasive)) ER/PR + HER2- ER/PR/HER2 + ER/PR- HER2 + TNBC or ‘ER-low + ’ Missing	785 (79%) 87 (9%) 43 (4%) 75 (8%) 9 (1%)
Index surgery (No. (%)) BCS Mastectomy	968 (81%) 222 (19%)
FIRST CEM SURVEILLANCE	
Age (mean, median (IQR) (range))	58.8, 59 (52–66) (27–92)
MD at first CEM (No. (%)) A B C D	69 (6%) 593 (50%) 437 (37%) 91 (8%)
BPE at first CEM (No. (%)) Minimal Mild Moderate Marked	686 (58%) 423 (36%) 68 (6%) 13 (1%)

*No* number, *DCIS* ductal carcinoma in situ, *ER* estrogen receptor, *PR* progesterone receptor, *HER2* human epidermal growth factor receptor 2, *TNBC* triple negative breast cancer, ‘*ER-low + *’ estrogen receptor low positive, *BCS* breast conserving surgery, *MD* mammographic density, *BPE* background parenchymal enhancement, *CEM* contrast-enhanced mammography

Outcomes from the first surveillance CEM have been reported previously: 6.1% of patients were recalled, 46.6% of recalls were TPs (PPV1 47%, PPV3 52%), CDR was 28.6/1000, and the contrast-directed TP recalls had pathology suggesting they were clinically significant [16].

### Follow-up surveillance episodes

**Fig. 1:**
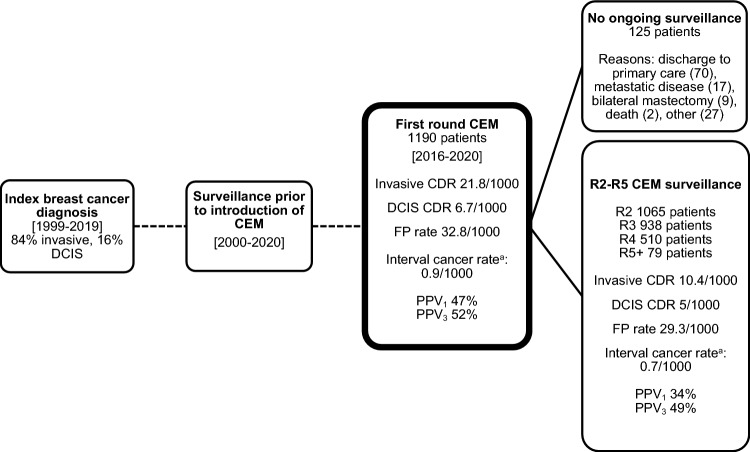
Study Flowchart, including key outcomes reported separately for first and subsequent round CEM surveillance. Combined figures are reported in various tables. *DCIS* ductal carcinoma in situ, *CEM* contrast-enhanced mammography, *CDR* cancer detection rate, *FP* false positive, *PPV*_*1*_ positive predictive value of any recall *PPV*_*3*_ positive predictive value of recalls resulting in biopsy, *R* round. ^a^Interval cancer rate defined as interval cancers per 1000 surveillance episodes detected following one surveillance episode and before the next scheduled surveillance episode

Until the end of 2022 there were 2,592 subsequent surveillance episodes for analysis (Fig. 1), including at least two episodes for 1,065/1,190 (89%) patients and at least three episodes for 938/1190 (79%) (Table 2). After the first CEM, 93% of subsequent surveillance imaging was contrast based (CEM and/or MRI). Reasons for imaging without contrast included patient choice, previous contrast reaction (4 patients, mild reactions), untreated hyperthyroidism, contrast extravasation and a worldwide contrast shortage in mid-2022. The median time between surveillance episodes was 12 months (interquartile range, IQR, 11–12 months).

**Table 2:** Surveillance episodes and cancer detection rate per round

Surveillance round	R1	R2	R3	R4 +	Total
No. patients	1190	1065	938	589	3782
Time since previous surveillance (months, median, IQR)	N/A	12 (11–12)	12 (11–12)	12 (11–12)	12 (11–12)
CDR (per 1000 episodes)	28.6	18.8	11.7	15.3	19.6
Contrast-based imaging (CEM and/or MRI, No., % of total)	1190 (100%)	995 (93%)	872 (93%)	539 (92%)	3596 (95%)
MRI only (no CEM, No., % of total)	0	3 (0.3%)	2 (0.2%)	8 (1%)	13 (0.3%)
MG ± US (No., % of total)	0	69 (6%)	65 (7%)	50 (8%)	184 (5%)
US alone (No., % of total)	0	1 (0.1%)	1 (0.1%)	0	2 (0.1%)

*N/A* not applicable, *R* round, *No* number, *CDR* cancer detection rate, *CEM* contrast-enhanced mammography, *MRI* magnetic resonance imaging, *MG* mammography, *US* ultrasound

Within the subsequent surveillance episodes, there were 116/2592 recalls (4.5%) of which 40/116 (34%) were TP and 76/116 (66%) were FP. The CDR was 15.4 per 1000 surveillance episodes, with PPV1 34% and PPV3 49% (Table 3).

**Table 3:** Cases recalled for further assessment, including contribution of contrast [16]

	First Surveillance CEM			Subsequent Surveillance CEM			Combined (all CEM episodes)		
Component of CEM leading to recall	2D ± C +	C + only	Any	2D ± C +	C + only	Any	2D ± C +	C + only	Any
Benign, FP (No. (% of recalls))	15 (47)	24 (59)	39 (53)	38 (62)	38 (69)	76 (66)	53 (57)	62 (65)	115 (61)
Malignant, TP (No. (% of recalls))	17 (53)	17 (41)	34 (47)	23 (38)	17 (31)	40 (34)	40 (43)	34 (35)	74 (39)
DCIS	3 (9)	5 (12)	8 (11)	5 (8)	8 (15)	13 (11)	8 (9)	13 (14)	21 (11)
Invasive	14 (44)	12 (29)	26 (36)	18 (30)	9 (16)	27 (23)	32 (34)	21 (22)	53 (28)
Total recalled (No. (%))	32 (3)	41 (3)	73 (6)	61 (2)	55 (2)	116 (4)	93 (2)	96 (3)	189 (5)
CDR (per 1000 episodes)	14.3	14.3	28.6	8.9	6.6	15.4	10.6	9.0	19.6
FPR (per 1000 episodes)	12.6	20.1	32.8	14.7	14.7	29.3	14	16.4	30.4
PPV_1_	53%	41%	47%	38%	31%	34%	43%	35%	39%
PPV_3_	53%	50%	52%	48%	50%	49%	50%	50%	50%

*No* number, *CEM* contrast-enhanced mammography, *2D* two dimensional, *C* + iodine image highlighting areas of contrast enhancement, *DCIS* ductal carcinoma in situ, *CDR* cancer detection rate, *FPR* false positive rate, *PPV*_1_ positive predictive value of any recall, *PPV*_3_ positive predictive value of recalls resulting in biopsy

Baseline characteristics of patients with TP and FP recalls were comparable (Table 4–5). Of the recalls, 17/40 (43%) surveillance-detected malignant lesions and 38/76 (50%) of FP recalls were contrast-directed. The contrast-directed PPV1 was 31% (PPV3 50%). Contrast-directed recalls identified an additional 17/40 malignant lesions, increasing the CDR from 8.9 to 15.4 per 1000 CEM episodes (*p* = 0.007), equivalent to a 73% increase in CDR with the use of contrast. Of all recalls, 82/116 (71%) required biopsy. Biopsies were US guided (22/82, 27%) or stereotactic (28/82, 34%) if the abnormality could be confidently visualised, and otherwise MRI guided (31/82, 38%). One recall required excisional biopsy to confirm benignity (1/82, 1%).

**Table 4:** Details of true positive recalls

		First Surveillance CEM				Subsequent Surveillance CEM				Combined (all CEM episodes)			
	Component of CEM leading to diagnosis	2D ± C +	C + only	Test for difference	Total	2D ± C +	C + only	Test for difference	Total	2D ± C +	C + only	Test for difference	Total
	Number of cases	17	17	N/A	34	23	17	N/A	40	40	34	N/A	74
Age	Median (range) Mean < 50 (N (%)) 50–59 (N (%)) 60–69 (N (%)) ≥ 70 (N (%))	66 (45–77) 63 1 (6) 6 (35) 5 (29) 5 (29)	57 (34–75) 56 5 (29) 4 (24) 5 (29) 3 (18)	t-test p = 0.320	61 (34–77) 59 6 (18) 10 (29) 10 (29) 8 (24)	59 (45–76) 60 2 (9) 10 (43) 8 (35) 3 (13)	58 (39–93) 61 4 (24) 5 (29) 3 (18) 5 (29)	t-test p = 0.261	59 (39–93) 60 6 (15) 15 (38) 11 (28) 8 (20)	61 (45–77) 61 3 (8) 16 (40) 13 (33) 8 (20)	58 (34–93) 57 9 (26) 9 (26) 8 (24) 8 (24)	t-test p = 0.165	59 (34–93) 58 12 (16) 25 (34) 21 (28) 16 (22)
Time since index cancer (years)	Median (range)) < 4 (N (%)) ≥ 4 (N (%))	3 (1–14) 9 (53) 8 (47)	3 (1–15) 10 (59) 7 (41)	t-test p = 0.500	3 (1–15) 19 (56) 15 (44)	4 (1–14) 8 (35) 15 (65)	4 (1–11) 8 (47) 9 (53)	t-test p = 0.522	4 (1–14) 16 (40) 24 (60)	4 (1–16) 17 (42) 23 (58)	3 (1–15) 18 (53) 16 (47)	t-test p = 0.484	4 (1–16) 35 (47) 39 (53)
Index Diagnosis (No. (%))	DCIS Invasive cancer	7 (41) 10 (59)	3 (18) 14 (82)	Fisher’s Exact p = 0.259	10 (29) 24 (71)	5 (22) 18 (78)	2 (12) 15 (88)	Fisher’s Exact p = 0.677	7 (18) 33 (82)	12 (30) 28 (70)	5 (15) 29 (85)	Fisher’s Exact p = 0.167	17 (23) 57 (77)
MD (No. (%))	A B C D	1 (6) 10 (59) 6 (35) 0 (0)	0 (0) 8 (47) 8 (47) 1 (6)	Fisher’s Exact p = 0.603	1 (3) 18 (53) 14 (41) 1 (3)	2 (9) 14 (61) 5 (22) 2 (9)	2 (12) 3 (18) 10 (59) 2 (12)	Fisher’s Exact p = 0.027	4 (10) 17 (43) 15 (38) 4 (10)	3 (8) 24 (60) 11 (28) 2 (5)	2 (6) 11 (32) 18 (53) 3 (9)	Fisher’s Exact p = 0.085	5 (7) 35 (47) 29 (39) 5 (7)
BPE (No. (%))	Nil/Minimal Mild Moderate Marked	7 (41) 8 (47) 1 (6) 1 (6)	10 (59) 6 (35) 1 (6) 0 (0)	Fisher’s Exact p = 0.732	17 (50) 14 (41) 2 (6) 1 (3)	14 (61) 5 (22) 1 (4) 3 (13)	5 (29) 9 (53) 3 (18) 0 (0)	Fisher’s Exact p = 0.026	19 (48) 14 (35) 4 (10) 3 (8)	21 (53) 13 (33) 2 (5) 4 (10)	15 (44) 15 (44) 4 (12) 0 (0)	Fisher’s Exact p = 0.164	36 (49) 28 (38) 6 (8) 4 (5)
Side (No. (%))	Ipsilateral Contralateral Bilateral	10 (59) 6 (35) 1 (6)	8 (47) 9 (53) 0 (0)	Fisher’s Exact p = 0.732	18 (53) 15 (44) 1 (3)	15 (65) 8 (35) 0 (0)	9 (53) 8 (47) 0 (0)	Fisher’s Exact p = 0.522	24 (60) 16 (40) 0 (0)	25 (63) 14 (35) 1 (3)	17 (50) 17 (50) 0 (0)	Fisher’s Exact p = 0.240	42 (57) 31 (42) 1 (1)
Morphology (No. (%))	DCIS Invasive	3 (18) 14 (82)	5 (29) 12 (71)	Fisher’s Exact p = 0.688	8 (24) 26 (76)	5 (22) 18 (78)	8 (47) 9 (53)	Fisher’s Exact p = 0.171	13 (32) 27 (68)	8 (20) 32 (80)	13 (38) 21 (62)	Fisher’s Exact p = 0.121	21 (28) 53 (72)
Size (mm, median (range))	Invasive cancer	16 (2–85)	19 (4–141)	t-test p = 0.871	18 (2–141)	13(0–25)	18 (1–100)	t-test p = 0.871	15 (1–100)	14 (0–85)	19 (1–141)	t-test p = 0.607	15 (0–141)
Size distribution invasive cancer (No. (%)	T1a T1b T1c T2 T3	2 (14) 3 (21) 3 (21) 5 (36) 1 (7)	1 (8) 3 (25) 3 (25) 4 (33) 1 (8)		3 (12) 6 (23) 6 (23) 9 (35) 2 (8)	5 (28) 2 (11) 6 (33) 5 (28) 0	1 (11) 2 (22) 4 (44) 1 (11) 1 (11)		6 (22) 4 (15) 10 (37) 6 (22) 1 (4)	7 (22) 5 (16) 9 (28) 10 (31) 1 (3)	2 (9) 5 (24) 7 (33) 5 (24) 2 (10)		9 (17) 10 (19) 16 (30) 15 (28) 3 (6)
Grade of Invasive cancer (No., (%))	1 2 3 Not stated^a^	1 (7) 7 (50) 5 (36) 1 (7)	2 (17) 3 (25) 7 (58) 0 (0)	Fisher’s Exact p = 0.679	3 (12) 10 (38) 12 (46) 1 (4)	1 (6) 8 (44) 6 (33) 3 (17)	0 (0) 4 (44) 4 (44) 1 (11)	Fisher’s Exact p = 1.000	1 (4) 12 (44) 10 (37) 4 (15)	2 (6) 15 (47) 11 (34) 4 (13)	2 (10) 7 (33) 11 (52) 1 (5)	Fisher’s Exact p = 0.501	4 (8) 22 (42) 22 (42) 5 (9)
Node + (%)		0	1 (8)	N/A	1 (4)	3 (17)	2 (22)	N/A	5 (19)	3 (9)	3 (14)	N/A	6 (11)
Phenotype of Invasive cancer (No. (%))	ER/PR + HER2- ER/PR/HER2 + ER/PR-HER2 + TNBC/’ER-low + ’ Unknown^b^	10 (71) 1 (7) 0 (0) 3 (21) 0 (0)	9 (75) 0 (0) 1 (8) 2 (17) 0 (0)	Fisher’s Exact p = 1.000	19 (73) 1 (4) 1 (4) 5 (19) 0 (0)	12 (67) 2 (11) 1 (6) 2 (11) 1 (6)	3 (33) 0 (0) 1 (11) 5 (56) 0 (0)	Fisher’s Exact p = 0.063	15 (56) 2 (7) 2 (7) 7 (26) 1 (4)	22 (69) 3 (9) 1 (3) 5 (16) 1 (3)	12 (57) 0 (0) 2 (10) 7 (33) 0 (0)	Fisher’s Exact p = 0.195	34 (64) 3 (6) 3 (6) 12 (23) 1 (2)
DCIS Size (mm, median, (range), mean)		33 (2–52) 29	20 (6–60) 25	t-test p = 0.84	21 (2–60) 27	20 (6–70) 27	12 (5–58) 17	t-test p = 0.43	15 (5–70) 21	23 (2–70) 28	15 (5–60) 20	t-test p = 0.44	18 (1.5–70) 23.1
Grade of DCIS (No. (%))	Low Int High	0 (0) 1 (33) 2 (67)	1 (20) 3 (60) 1 (20)	Fisher’s Exact p < 0.001	1 (13) 4 (50) 3 (38)	0 (0) 4 (80) 1 (20)	0 (0) 2 (25) 6 (75)	Fisher’s Exact p = 0.103	0 (0) 6 (46) 7 (54)	0 (0) 5 (63) 3 (38)	1 (8) 5 (38) 7 (54)	Fisher’s Exact p = 0.783	1 (5) 10 (48) 10 (48)
Breast surgery for recurrence (No. (%))	BCS TM None	9 (53) 7 (41) 1 (6)	8 (47) 8 (47) 1 (6)	Fisher’s Exact p = 1.000	17 (50) 15 (44) 2 (6)	10 (43) 13 (57) 0 (0)	5 (29) 12 (71) 0 (0)	Fisher’s Exact p = 0.745	15 (37) 25 (63) 0 (0)	19 (48) 20 (50) 1 (3)	13 (38) 20 (59) 1 (3)	Fisher’s Exact p = 0.481	32 (43) 40 (54) 2 (3)

*No* number, *CEM* contrast-enhanced mammography, *2D* two dimensional, *C* + iodine image highlighting areas of contrast enhancement, *DCIS* ductal carcinoma in situ, *MD* mammographic density, *BPE* background parenchymal enhancement, *mm* millimetre, + positive, *ER* estrogen receptor, *PR* progesterone receptor, *HER2* human epidermal growth factor receptor 2, *TNBC* triple negative breast cancer, ‘*ER-low* + ’ estrogen receptor low positive, immune immunohistochemistry, *BCS* breast conserving surgery, *TM* total mastectomy

^a^One case of malignant phyllodes, other cases too small to grade

^b^Too small to perform immunohistochemistry

**Table 5:** Details of false positive recalls from subsequent surveillance rounds

	Component of CEM leading to recall			Total
	2D ± C +	C + only	Test for difference	
Number of cases	38	38	N/A	76
Age–median (range) < 50 (No. (%)) 50–59 (No. (%)) 60–69 (No. (%)) ≥ 70 (No. (%))	56 4 (11) 20 (53) 9 (24) 5 (13)	58 5 (13) 18 (47) 10 (26) 5 (13)	t-test p = 0.970	56 9 (12) 38 (50) 19 (25) 10 (13)
Time since index cancer (years, median (range)) < 4 years (No. (%)) ≥ 4 years (No. (%))	3.5 19 (50) 19 (50)	5.5 11 (29) 27 (71)	t-test p = 0.100	4 (1–16) 30 (39) 46 (61)
Index morphology (No. (%)) DCIS Invasive cancer	9 (24) 29 (76)	9 (24) 29 (76)	Fisher’s Exact p = 1.00	18 (24) 58 (76)
MD (No. (%)) A B C D	5 (13) 20 (53) 10 (26) 3 (8)	0 (0) 17 (45) 14 (37) 7 (18)	Fisher’s Exact p = 0.06	5 (7) 37 (49) 24 (32) 10 (13)
BPE (No. (%)) Minimal Mild Moderate Marked	19 (50) 15 (39) 3 (8) 1 (3)	11 (29) 24 (63) 3 (8) 0 (0)	Fisher’s Exact p = 0.11	30 (39) 39 (51) 6 (8) 1 (1)
Cases biopsied (No. (%))	25 (65)	17 (45)	Fisher’s Exact p = 0.11	42 (55)
High risk lesions^a^ (No. (%))	1 (3)	2 (5)	N/A	3 (4)
Supplemental imaging for cases not biopsied (No. (%)) MRI US Spot tomography Early review CEM	3 (8) 4 (11) 3 (8) 3 (8)	14 (37) 0 (0) 0 (0) 7 (18)	Fisher’s Exact p = 0.001	17 (22) 4 (5) 3 (4) 10 (13)

*No* number, *CEM* contrast-enhanced mammography, *2D* two dimensional, *C* + iodine image highlighting areas of contrast enhancement, *DCIS* ductal carcinoma in situ, *MD* mammographic density, *BPE* background parenchymal enhancement, *MRI* magnetic resonance imaging, *US* ultrasound

^a^High risk lesions included atypical ductal hyperplasia, atypical lobular hyperplasia, lobular carcinoma in situ

### Cancer characteristics

Of the surveillance-detected malignant lesions, most (68%) were invasive; of these, the median size was 15mm (IQR 7–20mm), 37% were Grade 3, 19% were node-positive, and ER/PR + HER2- cancers were the most common (56%) followed by TNBC/’ER-low positive’ (26%) (Table 4). For patients with malignant lesions, 37% of patients had BCS (including 7/24 (29%) of those with ipsilateral malignant lesions) and 63% had mastectomy.

Around two-thirds (63%) of incident invasive cancers were the same subtype as the index cancer. For ipsilateral surveillance-detected invasive cancers, 67% were the same phenotype as the index cancer, 22% were a different phenotype, one case was too small for immunohistochemistry and for one case the index lesion was DCIS.

**Fig. 2:**
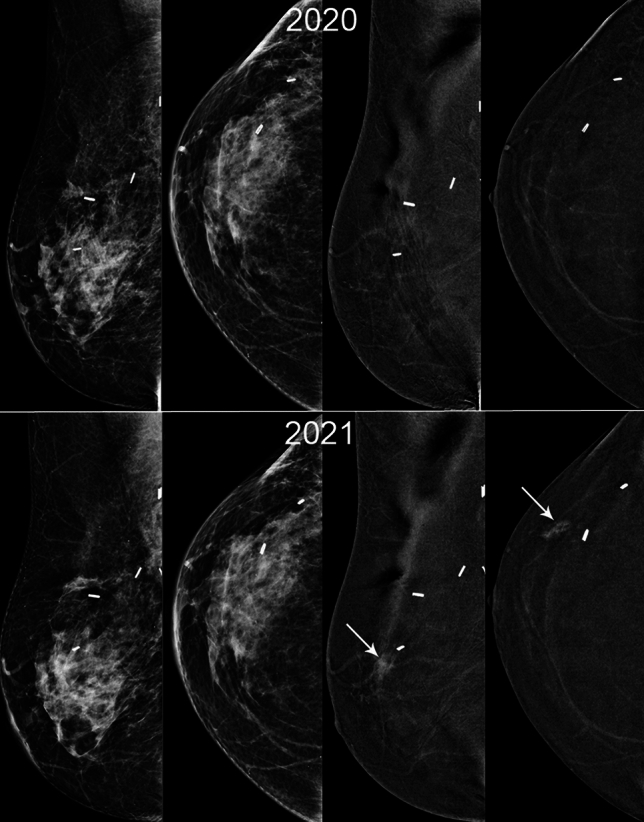
48F Index cancer 2017, 2.8cm TNBC right upper outer quadrant with positive nodes. PET no distant metastases. Treated with NACT with incomplete response. WLE clear margins, 2cm G3 TNBC, 2/29 positive nodes. Adjuvant radiotherapy. 3 surveillance rounds including first CEM 2020, clear. MD BIRADS B. New 19mm NME (white arrow) 2021 anterior to scar, seen on contrast only (2D/3D MG and US normal). MR biopsy 20mm TNBC G3 with LVI. Right Mastectomy

Malignant lesions detected after contrast-directed recall had comparable features to those identified on 2D alone or 2D and C + images in terms of size, grade and nodal status (Table 4). 5/9 (56%) contrast-directed TPs were TNBC or ‘ER-low positive’ (Fig. 2 illustrative case), with two of these patients having ER/PR + HER2- index cancers. Two TPs (2/40, 5%) had no contrast enhancement. One was a small cluster of calcifications with an incidental 1mm invasive lobular carcinoma. The other was clustered calcifications diagnosed as recurrent ipsilateral ER/PR/HER2 + invasive cancer 1 year following BCS and axillary dissection with previous pathological complete response to neoadjuvant systemic therapy. Both patients were on endocrine therapy at the time of recall.

Of the 76 FP recalls from the 2,592 surveillance episodes post initial CEM, 3 (4%) FP cases were high-risk lesions (atypical ductal hyperplasia, atypical lobular hyperplasia and lobular carcinoma in situ) (Table 5). 42/76 (55%) of FPs were biopsied; the remainder either had supplemental imaging or early review CEM which resolved the recall.

### Interval cancers

Two symptomatic interval cancers were identified, with a rate of 0.8 per 1000 surveillance screens (program sensitivity 96% (proportion of cancers detected through surveillance), with only 5% of all cancers presenting as interval cancers (Table 6). One was a contralateral Grade 3 ER/PR/HER2 + cancer that presented 10 months after routine surveillance CEM and 4 years following previous ER/PR + HER2-cancer. The other was an ipsilateral Grade 2 ER/PR- HER2 + recurrence with associated DCIS 8 months following first surveillance CEM and 15 months following BCS where the patient did not complete recommended adjuvant therapy. When the interval cancers were included, invasive cancers diagnosed in patients enrolled in surveillance were Stage 1, 2a, 2b and 3 in 72%, 14%, 10% and 3%, respectively.

**Table 6:** Details of interval cancers

Index cancer	Presentation	Number of previous surveillance CEMs	Time since last surveillance	Interval cancer
-2018 left IC NST G3 ER/PR + HER2-	Right breast symptoms	3	10 months	Right IC NST 34mm ER/PR/HER + , Node positive
-2018 right IC NST ER/PR- HER2 +	Right breast mass (at site of scar)	1	8 months	2019 right 50mm HG DCIS and 2 foci IC NST ER/PR-HER2 +

*CEM* contrast-enhanced mammography, *IC NST* invasive carcinoma of no special type, *G* grade, *ER* estrogen receptor, *PR* progesterone receptor, *HER2* human epidermal growth factor receptor 2, *mm* millimitre, *HG* high grade, *DCIS* ductal carcinoma in situ

### Risk factors

**Fig. 3:**
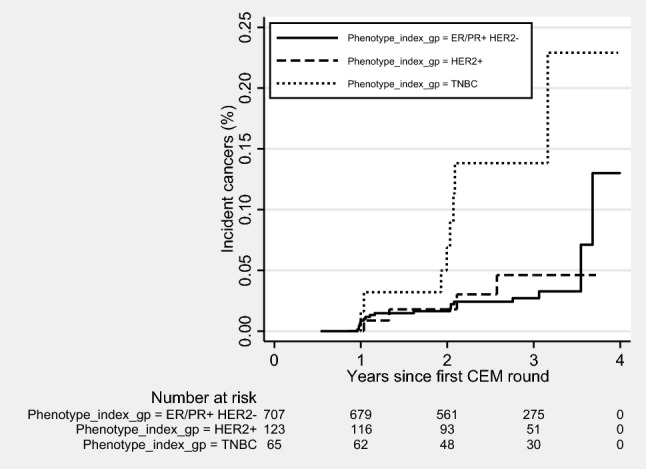
Incident invasive breast cancers according to index cancer phenotype and time since the first round of CEM surveillance. *CEM* contrast-enhanced mammography, *+*  positive, *ER* estrogen receptor, *PR* progesterone receptor, *HER2* human epidermal growth factor receptor 2, *TNBC* triple negative breast cancer

**Fig. 4:**
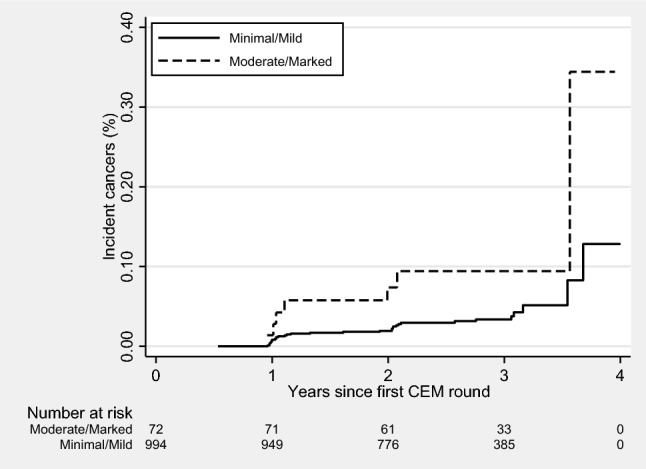
Incident invasive breast cancers according to baseline BPE and time since the first round of CEM surveillance. *BPE* background parenchymal enhancement, *CEM* contrast-enhanced mammography

Incident surveillance-detected cancers differed significantly by index cancer subtype (*χ*^*2*^ = 15.5, *p* = 0.0004, Fig. 3), with highest rates for patients with index TNBC. Incidence cancer rates were higher among the 6.9% of patients with moderate or marked BPE at first CEM surveillance episode (*χ*^*2*^ = 6.9, *p* = 0.009, Fig. 4), but did not differ significantly by age (*χ*^*2*^ = 4.4, *p* = 0.2).

There was a significant difference in contrast-directed recall rate based on breast density (Table 7). 528/1190 (46%) of women in this cohort had density C or D breasts. 12/17 (71%) of contrast-directed TP recalls were in women with density C or D breasts, compared with 5/17 (29%) in the 662/1190 (54%) of women with density A or B breasts (*p* < 0.001). 21/38 (55%) of contrast-directed FP recalls were in women with density C or D breasts, compared with 17/38 (45%) of women with density A or B breasts (*P* = 0.006).

**Table 7:** Recalls based on breast density

	All patients	TP: recall on 2D ± C +	TP: recall on C + only	Test for difference (TP)	FP: recall on 2D ± C +	FP: recall on C + only	Test for difference (FP)
MD (No. (%)) A B C D	69 (6) 593 (50) 437 (37) 91 (8)	2 (9) 14 (61) 5 (22) 2 (9)	2 (12) 3 (18) 10 (59) 2 (12)	Fisher’s Exact p = 0.027	5 (13) 20 (53) 10 (26) 3 (8)	0 (0) 17 (45) 14 (37) 7 (18)	Fisher’s Exact p = 0.06

*TP* true positive, *2D* two dimensional, *C* + iodine image highlighting areas of contrast enhancement, *FP* false positive, *MD* mammographic density

## Discussion

### Low interval cancer rate

Interval cancers in surveillance programs have previously been reported as more likely to be large, higher grade, receptor negative, and lymph node positive compared with cancers detected by surveillance imaging [23, 24]. As such, interval cancer rates are a key metric in surveillance programs. We report a very low rate of 0.8/1000 CEM surveillance episodes in women with a PHBC; this is markedly lower than interval cancer rates of 3.6 per 1000 MG surveillance episodes reported in large series of women with PHBC [1].

### Increased detection of clinically significant malignant lesions

Our findings are consistent with previous evidence that CEM for women with PHBC has a higher CDR than mammography alone [16, 25]. For subsequent rounds of CEM surveillance, there was a 73% increase in CDR with the use of contrast (contrast-directed TP recalls increased the CDR from 8.9 to 15.4 per 1000 surveillance episodes). With the initial CEM the CDR was 28.6/1000 screens and it remained high at 15.4/1000 screens in subsequent rounds. This is comparable to other studies assessing CEM and MRI in surveillance [10, 25–27] and supports ongoing use of CEM in surveillance. A reduction between prevalent and subsequent round cancer detection rates has also been seen in large screening studies using MRI in higher risk populations such as the DENSE trial [28].

The possibility of over-diagnosis must be considered with a more sensitive diagnostic modality. The data presented here are reassuring: 53% of malignant lesions detected after contrast-directed recall were invasive cancers, of which 89% were grade 2–3, two were node positive and 56% were TNBC or ‘ER-low positive’. The malignant lesions diagnosed after contrast-directed recall had characteristics comparable to those detected on 2D alone or 2D and C + images. The contrast-directed PPV3 was 50%.

The intent of surveillance imaging is to identify malignancies at an early stage when treatment may be more effective. In our series, invasive cancers diagnosed in patients with PHBC were Stage 1, 2A, 2B and 3 in 72%, 14%, 10% and 3% of cases. This compares with a large mammographic surveillance series reporting rates of 70%, 13%, 5% and 10%, respectively [1].

### Highly sensitive surveillance modality is warranted for women with PHBC

Improved survival rates have been shown when local recurrences and contralateral new primary cancers are detected on surveillance imaging rather than presenting symptomatically, supporting the use of sensitive imaging methods in women with PHBC [24, 29]. Whilst MRI is used in some higher risk groups, there would be substantial resource implications and access limitations should it be introduced as a standard. A 2020 meta-analysis of studies of MRI for surveillance in women with PHBC showed wide differences in MRI performance [12]. A US study of MRI within the Breast Cancer Surveillance Consortium reported increased cancer detection rates compared with mammography, but in contrast to our CEM study they found no difference in interval cancer rate [30]. A Korean study of MRI surveillance for young women reported a lower CDR (8.4/1000) than in our CEM study, potentially due to differences in the population [10, 31]. Many previous series have combined prevalent and incident rounds of surveillance, whereas our study evaluating incident CEM surveillance rounds provides more meaningful insights on ongoing surveillance [25].

High mammographic density is associated with a reduced sensitivity of mammography. In this cohort 12/17 (71%) of TP contrast-directed recalls were in the 46% of patients with BIRADS C or D density compared with 5/17 (29%) in the 54% with BIRADS A or B. This indicates that surveillance using CEM has a larger potential benefit in those with higher MD, while the benefit in those with lower MD is not trivial.

### Surveillance-detected malignant lesions differed by index cancer subtype and BPE

Adjuvant endocrine and/or radiation therapy likely contributed to the large proportion of women in this cohort (93%) with no, minimal or mild BPE. Cancer detection rates were higher among the 7% of women with moderate or marked BPE at first surveillance CEM. BPE is a recognised biomarker of breast cancer risk, particularly in high-risk populations [10, 32, 33]. As BPE may also mask a small cancer on CEM, MRI may offer an advantage in this population, as the enhancement kinetics with MRI may distinguish between background and pathological enhancement.

Surveillance-detected cancer rates also differed significantly by index cancer subtype with higher rates for women with index TNBC or ‘ER-low positive’ cancers. This is consistent with a report that patients with index TNBC or ER/PR-HER2 + cancer are at higher risk of locoregional recurrence [34].

This study adds to the body of evidence that BPE and index cancer subtype are important risk factors for subsequent breast malignancy and may inform decisions about surveillance modality and frequency. Whilst there is emerging evidence it may be safe to lengthen the surveillance interval for some women with PHBC [35], tailored surveillance should be considered, potentially extending the surveillance interval for those at lower risk, and continued annual surveillance with a sensitive modality such as CEM for those at higher risk.

### Acceptability and feasibility of routine CEM as surveillance

In this study there was good adherence to CEM as routine surveillance imaging. This contrasts with findings of MRI series where compliance reduces over time, and is consistent with surveys reporting a patient preference for CEM over MRI [10, 36]. When CEM was integrated into the radiology workflow, a key benefit at our institution was a 55% reduction in the use of bilateral breast US screening the following year [16]. Consistent with other CEM studies, contrast reactions were uncommon [37].

More than one third of biopsies required MRI guidance, and problem-solving MRI was used in half of FP cases not proceeding to biopsy, highlighting the need for MRI and MRI- or CEM-guided biopsy in any institution considering surveillance CEM. At initial surveillance CEM, 82% of FP recalls proceeded to biopsy, compared with only 55% for subsequent CEM rounds, likely due to the ability to compare sequential CEMs.

### Strengths and limitations of this study

Strengths of this study include a large cohort of patients with baseline CEM, with over 92% of patients having surveillance CEM for subsequent rounds, and details of all recalls. It is a heterogeneous surveillance population with a range of age and cancer subtypes. We have reported data from initial and subsequent CEM rounds separately to establish the baseline, subsequent and interval cancer rates, noting that many studies included in a 2020 meta-analysis of MRI for surveillance were criticised for combining prevalent and incident rounds [12].

Limitations of the study include a small number of patients having contrast-based imaging at the time of index diagnosis, and patients commencing surveillance CEM at various timepoints post index cancer diagnosis. This study does not currently include distant recurrence events, nor survival data, which will be important future work. The smaller proportion of invasive cancers diagnosed when Stage 2b or 3 is encouraging, but actual survival data are required to determine whether this translates to improved outcomes. Not all patients in our service had contrast imaging in surveillance, and we do not have the data to compare cancer detection and interval cancer rates for these women.

## Conclusions

This study shows that routine CEM for surveillance in women with PHBC is associated with persistently higher cancer detection rates, and interval cancer rates well below that in published series from mammographic surveillance. The pathology of the additional lesions identified and the low interval cancer rate indicate that the additional lesions are clinically significant. Future studies on the identification of risk factors for subsequent breast malignancy will be important to inform tailored surveillance.

## Data Availability

The datasets generated and analysed during this study are not publicly available due to potential patient identifiable data but are available from the corresponding author on reasonable request.
